# Association between waist-to-height ratio and osteoporosis in the National Health and Nutrition Examination Survey: a cross-sectional study

**DOI:** 10.3389/fmed.2024.1486611

**Published:** 2024-12-18

**Authors:** Hailong Li, Jianfeng Qiu, Zhe Gao, Chun Li, Jianjun Chu

**Affiliations:** ^1^Department of Orthopedics, The Second People’s Hospital of Fuyang, Fuyang, Anhui, China; ^2^School of Artificial Intelligence, Anhui University, Hefei, Anhui, China; ^3^Huaibei Miner General Hospital, Spinal and Trauma Surgery, Huaibei, Anhui, China; ^4^Department of Orthopedics, The First People’s Hospital of Hefei, The Third Affiliated Hospital of Anhui Medical University, Hefei, Anhui, China; ^5^Department of Orthopedics, The Second People’s Hospital of Hefei, Hefei Hospital Affiliated to Anhui Medical University, Hefei, Anhui, China

**Keywords:** waist-to-height ratio, osteoporosis, elderly people, NHANES, cross-sectional study

## Abstract

**Background:**

The link between waist-to-height ratio (WHtR) and osteoporosis (OP) remains a contentious issue in the field of medical research. Currently, the available evidence on this association is deemed insufficient. This topic has garnered significant attention and is a focal point of ongoing investigations.

**Methods:**

A retrospective cross-sectional study was conducted, involving 5,746 participants from the National Health and Nutrition Examination Survey. Data on various demographic and clinical parameters, including age, gender, race, poverty income ratio, educational level, smoking status, drinking status, cardiovascular disease, hypertension, diabetes mellitus, hemoglobin A1c, alanine transaminase, aspartate transaminase, serum total bilirubin, serum creatinine, uric acid, blood urea nitrogen, serum sodium, serum phosphorus, total calcium, serum potassium, and serum iron, were collected from all participants. The main analytical methods utilized in this study were multivariable logistic regression, restricted cubic splines, and threshold effect analysis to investigate the association between WHtR and OP.

**Results:**

A total of 5,746 elderly participants were enrolled, with a median age of 69.3 years. Compared with individuals with lower WHtR Q1 (≤0.36 to ≤0.56), the adjusted OR values for WHtR and OP in Q2 (<0.56 to ≤0.61), Q3 (<0.61 to ≤0.66), and Q4 (<0.66 to ≤ 0.94) were 0.63 (95% CI: 0.47–0.85, *p* = 0.003), 0.53 (95% CI: 0.37–0.76, *p* < 0.001), and 0.49 (95% CI: 0.35–0.68, *p* < 0.001), respectively. The association between WHtR and OP exhibited an L-shaped curve (nonlinear, *p* = 0.008) with an inflection point of roughly 0.57. The OR for the presence of OP was 0.50 (95% CI: 0.31–0.82, *p* = 0.007) in participants with WHtR <0.57. There was no association between WHtR and OP in participants with WHtR ≥0.57.

**Conclusion:**

The association between WHtR and OP showed an L-shaped curve, with an inflection point at around 0.57.

## 1 Introduction

Osteoporosis (OP) is a disease defined by a decrease in bone mass and density, resulting in diminished bone strength and an increased risk of fractures, particularly in the hip and spine. The incidence of OP in the United States is approximately 12.6% among persons aged 50 years and older (1). Currently, due to the continuous advancement of the worldwide population aging, the annual number of patients suffering from fractures caused by OP is as high as 8.9 million (2), indicating that there is one individual with OP who experiences fractures every 3 s (3). The significant medical and societal impact of this issue has generated widespread public attention, highlighting the crucial need to investigate the factors associate with OP in order to prevent its occurrence.

Numerous osteoporosis risk factors have been proposed in previous studies, such as age, gender, obesity, smoking, alcohol consumption, calcium intake, physical activity, and others. Among these, obesity is particularly significant due to its complex interactions with bone metabolism (4). Assessment of obesity, particularly central obesity, is crucial in understanding its relationship with OP. Common indicators include body mass index (BMI), waist circumference (WC), waist-to-hip ratio (WHR), and waist-to-height ratio (WHtR). While BMI provides a general measure of obesity, WC, WHR, and WHtR offer insights into the distribution of fat, with abdominal fat being more closely related to metabolic complications and bone health (5). Central obesity is characterized by excessive fat accumulation around the abdomen and can have an impact on bone health through chronic inflammation, hormonal changes, and increased mechanical loading (6). In addition, when evaluating central obesity, WC and WHR may be influenced by factors such as gender or ethnicity, whereas the WHtR is comparatively less impacted (7). Therefore, the WHtR is the most pragmatic approach for evaluating abdominal obesity (8). However, the relationship between WHtR and OP remains ambiguous (4, 5, 9). Thus, it is highly meaningful to investigate the association between WHtR and OP across different demographic groups in order to assess if the WHtR and the OP were correlated. The study aims to explore the association between WHtR and OP.

To achieve these objectives, a cross-sectional research was conducted which involved elderly people from the National Health and Nutrition Examination Survey (NHANES). This research design enables the investigation of the relationships between WHtR and OP within a substantial and varied sample group.

## 2 Method

### 2.1 Data source

This cross-sectional study employed NHANES data from 2005 to 2010, 2013 to 2014, and 2017 to 2018, which was conducted by the Centers for Disease Control and Prevention (10). The objective of the NHANES project was to evaluate the health and nutritional status of non-institutionalized Americans using a stratified multistage probability survey (11). The NHANES collects demographic and in-depth health information via home visits, screening, and laboratory testing conducted by a mobile examination center (MEC). The NHANES was authorized by the National Center for Health Statistics (NCHS) Ethics Review Committee, and all participants completed written informed consent forms before participation. The secondary analysis did not require additional Institutional Review Board approval (12). The NHANES data are accessible via the NHANES website.^1^ The study population consisted of individuals aged 60 years and older who had completed an interview. Individuals with incomplete data on variables such as stand height, waist circumference, bone density assessment, or covariates were excluded from the analysis.

### 2.2 Determination of BMD and the diagnosis of OP

The bone mineral density (BMD) levels were measured in several areas (total femur, femur neck, and lumbar spine) in the NHANES study using dual-energy X-ray absorptiometry (DXA) with Hologic QDR-4500A fan-beam densitometers (Hologic, Inc., Bedford, Massachusetts). The left hip was typically examined, while the right hip was only imaged in cases where the patient reported a fracture, pin, or replacement in the left hip. The BMD of the lumbar spine was determined by averaging the BMD measurements obtained from the first to fourth vertebrae in the lumbar region. Pregnant patients, individuals with a history of radiographic contrast material, those with bilateral hip fractures, replacements, or pins, and those exceeding 450 lbs. in weight were excluded from the DXA examination. In this study, the diagnosis of OP was established by referencing relevant literature (13, 14) and employing established methods to convert the BMD levels of the femoral neck and lumbar spine into *T*-scores. Individuals were classified as suffering from OP, osteopenia, or having normal bone density according to the *T*-score: ≤ −2.5, −2.5 < *T*-score ≤ −1, and *T*-score > −1, respectively. Patients with osteopenia and those with normal bone density were classified as non-OP.

### 2.3 Waist-to-height ratio (WHtR)

The WHtR was determined by dividing the waist circumference by the standing height of the participant.

### 2.4 Covariate assessment

Drawing from existing research and clinical insights, the selection of covariates in this study primarily encompasses demographic variables, metabolic and chronic disease states, along with biochemical parameters.

The modified model includes demographic characteristics. Gretl Hendrickx et al. have asserted that age and gender correlate with osteoporosis (15). Relevant investigations have also substantiated the connection between ethnicity, poverty-income ratio (PIR), and educational attainment with bone mineral density (16–18). Lifestyle variables such as smoking and alcohol intake affect the incidence of osteoporosis (15). A literature study by Mahmood Safaei et al. demonstrates that age, gender, ethnicity, PIR, educational attainment, smoking, and alcohol intake are factors of obesity (19). Consequently, these demographic determinants have a dual function in the etiology of osteoporosis and obesity, highlighting their importance in the updated model.

Previous research has demonstrated a correlation between obesity and osteoporosis in individuals with metabolic and chronic illnesses. A study by Bo Wu et al. showed that diabetes mellitus (DM) may result in secondary osteoporosis (20). A review of the literature by Michel et al. also found that people who have had osteoporotic fractures or low bone mineral density are more likely to have coronary artery disease and stroke than people who do not have osteoporosis. In contrast, those with cardiovascular disorders have a heightened risk of bone loss and osteoporotic fractures (21). Cappuccio FP et al. indicated that raised blood pressure in older white women is associated with greater femoral neck bone loss, suggesting a linkage between osteoporosis and hypertension (22). Research indicates that obesity correlates with a heightened risk of several chronic and metabolic disorders, such as asthma, diabetes, hypertension, and cardiovascular diseases (CVD) (19).

Adjustments were made to certain biochemical markers in the model for the following reasons: HoJeongDo et al. indicated that liver enzymes, including alanine aminotransferase (ALT) and aspartate aminotransferase (AST), have a negative correlation with bone mineral density (BMD) (23). Liu Chenbin et al. elucidated the relationship between obesity and liver enzymes (AST, ALT) (24). Researchers have recorded a correlation between total bilirubin and BMD (25). Zara Jenko-Plaznikar et al. found a decrease in blood bilirubin concentrations in overweight healthy individuals and a negative correlation with abdominal obesity (26). Additionally, studies employing Mendelian randomization revealed a robust correlation between glycated hemoglobin and estimated bone mineral density (eBMD), potentially facilitated by non-glycemic pathways such as red blood cell indices (27). A research study found that lifestyle weight reduction programs effectively improve glycated hemoglobin levels in individuals with type 2 diabetes mellitus (T2DM) across various ethnic groups (28). In addition, UgurA et al.’s study on biochemical markers related to renal function found that high blood urea nitrogen levels are a sign of lower bone mineral density in the femoral neck (29). Previous investigations have also revealed a correlation between obesity and urea nitrogen (30). Guan Yu et al. identified a favorable connection between serum creatinine and BMD in elderly Chinese individuals with normal renal function (31). Mehl Rubin et al.’s research reveals that obese women have elevated average serum creatinine and creatinine clearance rates compared to healthy normal-weight women (32). The investigation by Yan Dandan et al. showed that uric acid has a protective influence on bone metabolism in Chinese postmenopausal women (33). A research study indicated that elevated blood uric acid (SUA) levels correlate with a heightened risk of obesity (34). Moreover, prior research has shown correlations between obesity and osteoporosis with blood electrolytes, including sodium, potassium, iron, phosphorus, and total calcium (35–42). Wang et al. further showed that sufficient vitamin D consumption decreases the incidence of osteoporosis in postmenopausal women in the United States, particularly in those aged 65 and above (43). Hajhashmi et al. conducted a meta-analysis that revealed a negative correlation between blood vitamin D levels and the incidence of abdominal obesity in adults (44).

NHANES researchers developed standardized questionnaires to gather demographic data, such as gender (male or female), age, PIR, race (non-Hispanic White, non-Hispanic Black, Mexican American, other Hispanic, or other race, including multi-racial), and education level (less than high school: less than 9th grade and 9-11th grade (including 12th grade with no diploma); high school or equivalent: high school graduate/GED or equivalent; above high school: some college or AA degree and college graduate or above). You can classify smoking status into three categories: never (less than 100 cigarettes), former (more than 100 cigarettes but stopped), and current (more than 100 cigarettes and still smoke). Drinking status (never: drank less than 12 drinks in a lifetime; former: drank more than 12 drinks in 1 year but abstained from alcohol the previous year, or more than 12 drinks overall) Currently, heavy alcohol consumption is defined as having three or more drinks per day for women, four drinks per day for men, or binge drinking on five or more days per month (four drinks on the same occasion for women, five drinks on the same occasion for men). Presently, moderate alcohol consumption is defined as two or more drinks for women, three for men, or binge drinking for more than 2 days per month. Currently, mild alcohol consumption is defined as ≤1 drink for women and ≤ 2 drinks for men per day. The self-reported CVD history included previous diagnoses of heart failure, coronary heart disease, angina, heart attack, or stroke. In order to calculate the mean blood pressure, disregard diastolic measurements of zero unless all diastolic measurements are zero. We considered a single reading as the mean value. To calculate several readings, do not include the first reading in the computation. The diagnosis of hypertension occurred when the systolic blood pressure reached or exceeded 140 mmHg or when the diastolic blood pressure reached or exceeded 90 mmHg. A doctor’s diagnosis, glycohemoglobin (HbA1c) values >6.5%, fasting glucose levels ≥7.0 mmol/L, blood glucose levels ≥11.1 mmol/L from a random 2-h oral glucose tolerance test, or the use of diabetes medicine or insulin are the diagnostic criteria for DM. We followed standardized protocols to measure the following: hemoglobin A1c (HbA1c), ALT, AST, serum total bilirubin, serum creatinine, uric acid, blood urea nitrogen (BUN), serum sodium, serum phosphorus, total calcium, serum potassium, and serum iron. The NHANES website provides further information.

### 2.5 Statistical analysis

In accordance with the NHANES analytic criteria, the current study accounted for intricate sample designs and sampling weights (45). A weighted analysis using Wtmec2 year weights was conducted. We included data from NHANES for the years 2005–2010, 2013–2014, and 2017–2018. In this research, the sample weights for data analysis were computed in the following manner: The sampling weight was calculated as 1/5 × wtmec2yr.

Categorical variables are shown as unweighted counts (weighted percentages), whereas continuous variables are expressed using the mean (standard deviation, SD) or median (interquartile range, IQR), as applicable. One-way analyses of variance were used for data with a normal distribution, Kruskal-Wallis tests were used for data with a skewed distribution, and chi-square tests were used for categorical variables to look at the differences between the groups. Logistic regression models were used to ascertain the odds ratios (OR) and 95 percent confidence intervals (95% CIs) for the association between WHtR and OP. Our model adjustment was based on the following principles: Model 1 included variables, such as sex and uric acid, whose effect values changed by more than 10%. Model 2 was adjusted for adding variables with *p*-values below 0.05 in the univariate analysis based on model 1, thus further including age, race, PIR, educational level, smoking status, drinking status, DM, HbA1c, ALT, and serum total bilirubin and serum phosphorus. Model 3 was the fully adjusted model, which also included adjustments for CVD, hypertension, AST, serum creatinine, BUN, serum sodium, total calcium, serum potassium, and serum iron, all based on Model 2. Furthermore, a restricted cubic spline (RCS) regression analysis was conducted using 3 knots as recommended by Harrell (46). This analysis aimed to evaluate the linearity and investigate the dose–response relationship between WHtR and OP while controlling for other factors in Model 3. To identify the threshold effect, we have enhanced a two-piecewise linear regression model based on the smoothing curve. This model also takes into account possible confounders in Model 3.

Interactions and stratified analyses were performed based on gender (male vs. female), race (Hispanic vs. non-Hispanic), education level (≤12y vs. >12y), smoking status (never vs. former or current), drinking status (never vs. former or current), and diabetes mellitus (DM) (yes vs. no). The study used logistic regression models to measure heterogeneity among the subgroups as well as likelihood ratio testing to examine their interactions. In order to assess the strength and reliability of our findings, we further included patients who had data on blood total 25-hydroxyvitamin D for sensitivity analyses.

All of these models’ computed effect sizes and *p* values were presented and contrasted. R Statistical Software (Version 4.2.2, The R Foundation)^2^ and the Free Statistics analytic platform (Version 1.9, Beijing, China)^3^ (47) were used for all analyses. FreeStatistics is a software suite that offers user-friendly interfaces for doing common analyses and visualizing data. R serves as the statistical engine, and Python implements the graphical user interface (GUI). It was specifically created for doing analysis that can be replicated and for engaging in computing that allows for interaction. A two-sided *p* value less than 0.05 was deemed to be statistically significant.

## 3 Results

### 3.1 Study population

Of the 19,087 participants of the NHANES 2005–2018 aged ≥60y, 13341were excluded for the following reasons:

- Missing data on body measures (*n* = 2,907).
- Missing data on OP detection (*n* = 8,781).
- Missing data on covariates (*n* = 1,653).

**Figure 1:**
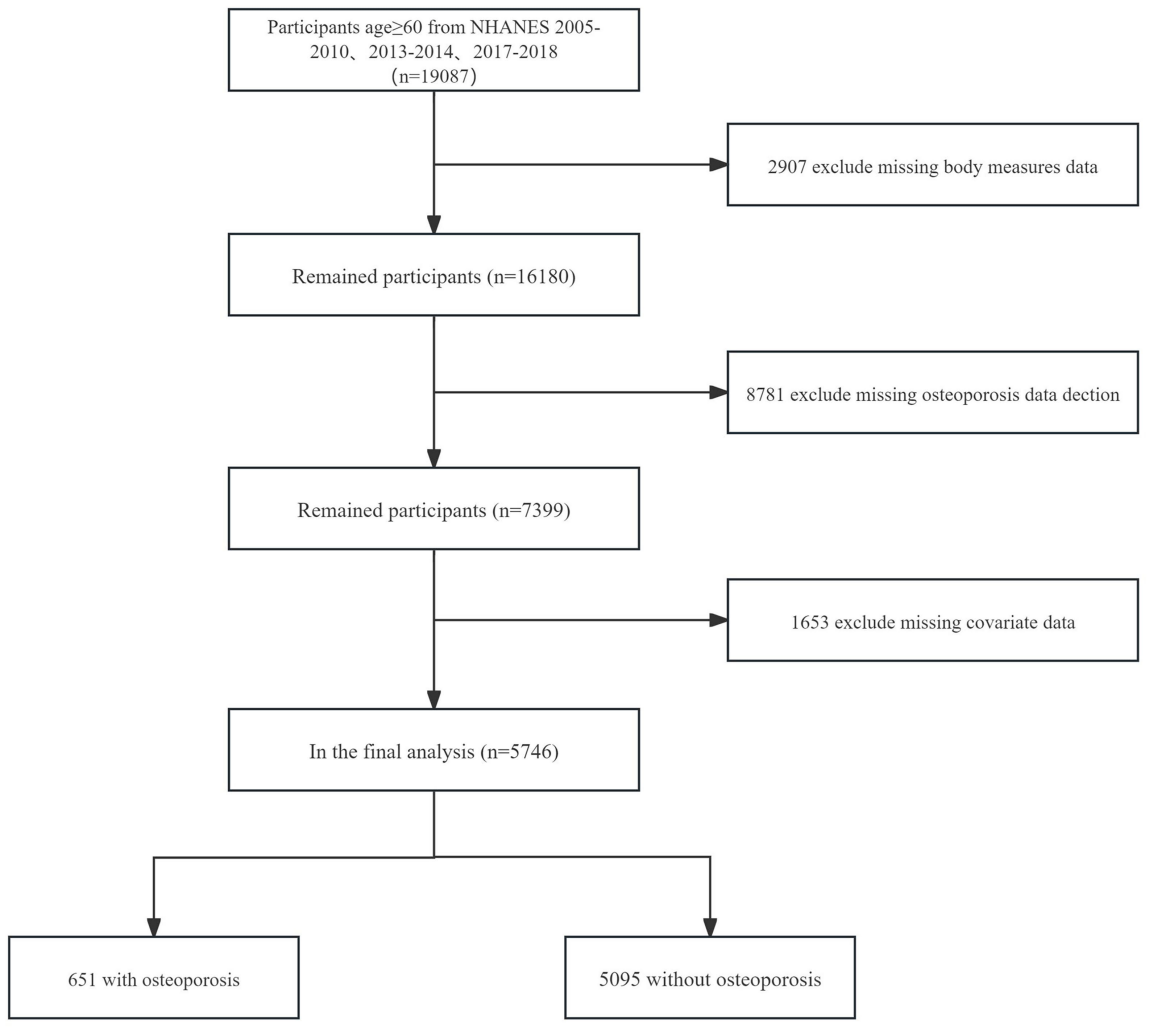
The study flow diagram.

Thus, 5,746 participants were included in the analysis (Figure 1).

### 3.2 Baseline characteristics

Table 1 displays the fundamental characteristics of the 5,746 individuals included in the research, categorized based on their WHtR quartiles. A total of 651 people, or 11% of the population, had OP. The mean age of the research participants was 69.32 (6.80) years, and 2,742 (53.29%) people were female. Compared to those with lower WHtR, those with higher WHtR were more likely to be non-Hispanic White, have lower family income, and be more likely to be female. They also had lower current smoking and drinking rates, as well as a higher prevalence of hypertension, diabetes, and CVD; had lower levels of serum iron; and had higher levels of HbA1c, uric acid, ALT, AST, and BUN.

**Table 1:** Population characteristics by categories of WHtR.

Characteristic	Waist-to-height ratio					
	Total	Q1 [0.36, 0.56]	Q2 [0.56, 0.61]	Q3 [0.61, 0.66]	Q4 [0.66, 0.94]	*p*-value
NO.	5,746	1,437	1,436	1,436	1,437	
Age (year), Mean (SD)	69.32 (6.80)	69.42 (7.03)	69.47 (6.86)	69.53 (6.78)	68.86 (6.52)	0.147
Gender, *n* (%)						
Male	3,004 (46.71)	767 (41.83)	817 (51.91)	795 (52.23)	625 (41.15)	<0.001
Female	2,742 (53.29)	670 (58.17)	619 (48.09)	641 (47.77)	812 (58.85)	
Race, *n* (%)						
Non-Hispanic White	3,157 (81.13)	795 (80.20)	783 (80.80)	785 (81.22)	794 (82.33)	<0.001
Non-Hispanic Black	1,074 (7.56)	322 (8.32)	254 (7.25)	242 (7.01)	256 (7.62)	
Mexican American	729 (3.97)	106 (2.23)	178 (3.97)	221 (4.90)	224 (4.83)	
Other Hispanic	441 (2.99)	71 (2.04)	114 (3.31)	127 (3.35)	129 (3.31)	
Other Race^*^	345 (4.35)	143 (7.21)	107 (4.67)	61 (3.52)	34 (1.90)	
PIR, Mean (SD)	3.13 (1.54)	3.27 (1.56)	3.18 (1.55)	3.1 0 (1.54)	2.96 (1.50)	0.002
Education level, *n* (%)						
Less than high school	1,656 (18.39)	359 (15.66)	415 (18.93)	420 (18.80)	462 (20.24)	0.003
High school or equivalent	1,417 (26.11)	325 (22.29)	348 (26.97)	383 (27.98)	361 (27.32)	
Above high school	2,673 (55.50)	753 (62.05)	673 (54.10)	633 (53.22)	614 (52.44)	
Smoking status, *n* (%)						
Never	2,706 (48.18)	679 (51.06)	695 (50.27)	664 (45.0 9)	668 (46.16)	<0.001
Former	2,309 (40.89)	498 (35.23)	558 (37.75)	630 (45.43)	623 (45.35)	
Now	731 (10.94)	260 (13.71)	183 (11.98)	142 (9.48)	146 (8.49)	
Drinking status, *n* (%)						
Never	930 (13.81)	220 (13.10)	232 (14.35)	236 (13.73)	242 (14.08)	0.019
Former	1,382 (19.86)	314 (17.11)	330 (18.62)	359 (22.61)	379 (21.20)	
Mild	2,377 (46.80)	659 (51.87)	600 (48.19)	574 (42.96)	544 (44.00)	
Moderate	620 (12.49)	155 (12.37)	152 (11.97)	154 (11.57)	159 (14.01)	
Heavy	437 (7.05)	89 (5.55)	122 (6.88)	113 (9.12)	113 (6.72)	
CVD, *n* (%)						
No	4,443 (78.35)	1,162 (83.00)	1,146 (80.91)	1,077 (75.96)	1,058 (73.39)	<0.001
Yes	1,303 (21.65)	275 (17.00)	290 (19.09)	359 (24.04)	379 (26.61)	
Hypertension, *n* (%)						
No	1767 (33.50)	597 (45.78)	467 (36.38)	409 (30.59)	294 (20.94)	<0.001
Yes	3,979 (66.50)	840 (54.22)	969 (63.62)	1,027 (69.41)	1,143 (79.06)	
DM, *n* (%)						
No	3,991 (74.04)	1,205 (88.65)	1,057 (79.05)	933 (69.78)	796 (58.30)	<0.001
Yes	1755 (25.96)	232 (11.35)	379 (20.95)	503 (30.22)	641 (41.70)	
HbA1c(%), Mean (SD)	5.89 (0.90)	5.63 (0.68)	5.81 (0.82)	5.97 (0.94)	6.16 (1.05)	<0.001
Uric acid (mg/dl), Mean (SD)	5.64 (1.41)	5.10 (1.35)	5.55 (1.33)	5.84 (1.35)	6.07 (1.41)	<0.001
Serum sodium (mmol/L), Mean (SD)	139.71 (2.58)	139.72 (2.57)	139.61 (2.55)	139.87 (2.56)	139.65 (2.64)	0.385
Serum phosphorus (mg/dl) Mean (SD)	3.75 (0.55)	3.81 (0.57)	3.75 (0.53)	3.71 (0.54)	3.71 (0.55)	<0.001
Total calcium (mg/dl), Mean (SD)	9.45 (0.37)	9.46 (0.36)	9.47 (0.37)	9.44 (0.37)	9.44 (0.39)	0.258
Serum potassium (mmol/L), Mean (SD)	4.09 (0.38)	4.07 (0.38)	4.08 (0.36)	4.09 (0.39)	4.10 (0.40)	0.617
Serum iron (ug/dl), Mean (SD)	86.55 (31.39)	90.14 (32.94)	87.60 (31.52)	87.95 (31.26)	80.49 (28.87)	<0.001
ALT (u/L), Median [IQR]	20.00 [16.00, 25.39]	19.00 [16.00,24.00]	20.00 [16.00,25.00]	20.00 [16.00,26.00]	21.00 [17.00,27.00]	0.001
AST (u/L), Mean (SD)	24.92 (11.07)	25.35 (8.28)	24.66 (8.23)	24.14 (8.54)	25.48 (16.72)	0.007
Serum total bilirubin(mg/dl), Mean (SD)	0.70 (0.30)	0.73 (0.30)	0.71 (0.28)	0.70 (0.29)	0.66 (0.32)	0.002
Serum creatinine (mg/dl), Mean (SD)	0.98 (0.41)	0.96 (0.46)	0.98 (0.33)	1.01 (0.46)	0.99 (0.38)	0.004
BUN (mg/dl), Mean (SD)	16.42 (6.41)	15.96 (6.01)	16.04 (5.59)	16.78 (6.32)	16.91 (7.51)	0.001
Osteoporosis, *n* (%)						
No	5,095 (87.93)	1,180 (81.34)	1,282 (88.72)	1,311 (90.48)	1,322 (91.40)	<0.001
Yes	651 (12.07)	257 (18.66)	154 (11.28)	125 (9.52)	115 (8.60)	

WHtR, waist to height ratio; Q, quartiles; * Other not defined by the NHANES but it does include multi-racial and non-Hispanic Asian; PIR, poverty-to-income ratio; CVD, Cardiovascular Disease; DM, diabetes mellitus, HbA1c, hemoglobin A1c; ALT, alanine transaminase; AST, alanine transaminase; BUN, blood urea nitrogen.

### 3.3 Relationship between WHtR and osteoporosis

The univariate analysis revealed that age, gender, race, education level, smoke status, drink status, DM, HbA1c, Alt, total bilirubin, uric acid, and serum phosphorus were all shown to be associated with OP, as indicated by Table 2.

**Table 2:** Association of covariates and osteoporosis risk.

Variables	OR (95% CI)	*p*_value
Age	1.09 (1.07, 1.11)	<0.001
Gender		
Male	1 (reference)	
Female	4.67 (3.68, 5.94)	<0.001
Race		
Non-Hispanic White	1 (reference)	
Non-Hispanic Black	0.35 (0.24, 0.50)	<0.001
Mexican American	0.84 (0.61, 1.15)	0.265
Other Hispanic	1.03 (0.69, 1.53)	0.902
Other Race^*^	1.46 (1.05, 2.04)	0.024
PIR	0.79 (0.73, 0.85)	<0.001
Education level		
Less than high school	1 (reference)	
High school or equivalent	0.80 (0.61, 1.05)	0.100
Above high school	0.60 (0.47, 0.75)	<0.001
Smoking status		
Never	1 (reference)	
Former	0.59 (0.47, 0.74)	<0.001
Now	1.15 (0.83, 1.59)	0.383
Drinking status		
Never	1 (reference)	
Former	0.67 (0.51,0.87)	0.004
Mild	0.47 (0.36,0.61)	<0.001
Moderate	0.42 (0.26,0.68)	<0.001
Heavy	0.71 (0.44,1.14)	0.151
CVD		
No	1 (reference)	
Yes	1.19 (0.93,1.53)	0.173
Hypertension		
No	1 (reference)	
Yes	0.93 (0.73,1.18)	0.545
DM		
No	1 (reference)	
Yes	0.71 (0.55,0.93)	0.013
HbA1c (%)	0.80 (0.71,0.91)	0.001
ALT (u/L)	0.97 (0.95,0.98)	<0.001
AST (u/L)	1.0 (0.99,1.01)	0.562
Serum total bilirubin(mg/dl)	0.48 (0.31,0.75)	0.001
Serum creatinine (mg/dl)	0.90 (0.68,1.20)	0.464
Uric acid (mg/dl)	0.74 (0.68,0.82)	<0.001
BUN (mg/dl)	1.01 (0.99,1.02)	0.233
Serum sodium (mmol/L)	1.01 (0.98,1.05)	0.524
Serum phosphorus (mg/dl)	1.92 (1.58,2.33)	<0.001
total calcium (mg/dl)	0.84 (0.63,1.13)	0.257
Serum potassium (mmol/L)	1.24 (0.93,1.64)	0.135
Serum iron (ug/dl)	1.0 (0.99,1.00)	0.064

Q, quartiles; OR, odds ratio; CI, confidence interval; * Other not defined by The NHANES but it does include multi-racial and non-Hispanic Asian; PIR, poverty-to-income ratio; CVD, Cardiovascular Disease; DM, diabetes mellitus; HbA1c, hemoglobin A1c; ALT, alanine transaminase; AST, alanine transaminase; BUN, blood urea nitrogen.

**Figure 2:**
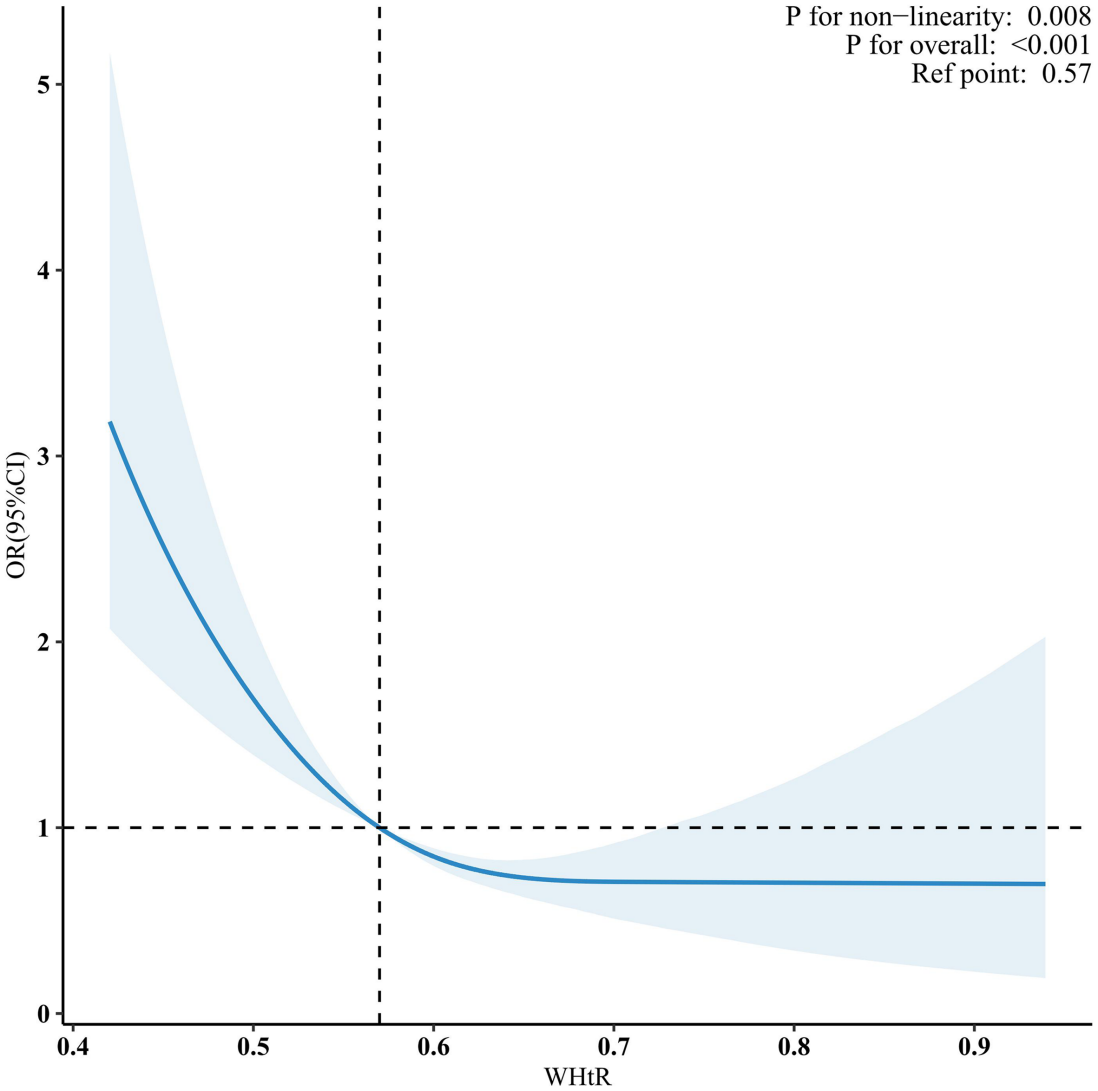
Nonlinear dose–response relationship between WHtR and OP. Solid and dashed lines indicate the predicted value and 95% CI. WHtR, waist to height ratio; OP, osteoporosis. The restricted cubic spline model was adjusted for age, gender, race, poverty income ratio (PIR), educational level, smoking status, drinking status, cardiovascular disease (CVD), hypertension, diabetes mellitus (DM), hemoglobin A1c (HbA1c), alanine transaminase (ALT), aspartate transaminase (AST), serum total bilirubin, serum creatinine, uric acid, blood urea nitrogen (BUN), serum sodium, serum phosphorus, total calcium, serum potassium, serum iron.

When the WHtR was examined using quartiles, a significant negative correlation was seen between WHtR and OP after accounting for any confounding factors. When comparing individuals with a lower WHtR in the range of Q1 (≤0.36 to ≤0.56), the adjusted odds ratio (OR) values for WHtR and OP in the Q2 range (<0.56 to ≤0.61), Q3 range (<0.61 to ≤0.66), and Q4 range (<0.66 to ≤0.94) were 0.63 (95% CI: 0.47–0.85, *p* = 0.003), 0.53 (95% CI: 0.37–0.76, *p* < 0.001), and 0.49 (95% CI: 0.35–0.68, *p* < 0.001), respectively, as shown in Table 3. The relationship between WHtR and OP showed an L-shaped curve, indicating a nonlinear correlation (*p* < 0.001) in restricted cubic splines (RCS) analysis (Figure 2). In the threshold analysis, the odds ratio (OR) for developing OP was 0.50 (95% confidence interval [CI]: 0.31–0.82, *p* = 0.007) among patients with WHtR less than 0.57 (Table 4). With each 0.1 rise in WHtR, the chance of OP is lowered by 50%.

**Table 3:** Association between WHtR and osteoporosis.

WHtR	No.	Crude OR (95%CI)	*p-*value	Model 1 OR (95% CI)	*p*-value	Model 2 OR (95% CI)	*p*-value	Model 3 OR (95% CI)	*p*-value
Q1 [0.36, 0.56]	1,437	1 (Ref)		1 (Ref)		1 (Ref)		1 (Ref)	
Q2 [0.56,0.61]	1,436	0.55 (0.43, 0.72)	<0.001	0.64 (0.48, 0.84)	0.002	0.61 (0.45, 0.82)	0.001	0.63 (0.47, 0.85)	0.003
Q3 [0.61,0.66]	1,436	0.46 (0.33, 0.63)	<0.001	0.54 (0.39, 0.75)	<0.001	0.54 (0.38, 0.76)	<0.001	0.53 (0.37, 0.76)	<0.001
Q4 [0.66,0.94]	1,437	0.41 (0.30, 0.55)	<0.001	0.42 (0.31, 0.58)	<0.001	0.49 (0.35, 0.68)	<0.001	0.49 (0.35, 0.68)	<0.001
Trend test			<0.001		<0.001		<0.001		<0.001

WHtR, waist to height ratio; Q, quartiles; OR, odds ratio; CI, confidence interval; Ref, reference.

Crude: no other covariates were adjusted.

Model 1 was adjusted for gender, uric acid.

Model 2 was adjusted for model1 + age, race, poverty income ratio (PIR), educational level, smoking status, drinking status, diabetes mellitus (DM), hemoglobin A1c (HbA1c), alanine transaminase (ALT), serum total bilirubin, serum phosphorus.

Model 3 was adjusted for model2 + cardiovascular disease (CVD), hypertension, aspartate transaminase (AST), serum creatinine, blood urea nitrogen (BUN), serum sodium, total calcium, serum potassim, serum iron.

**Table 4:** Threshold effect analysis of the relationship of WHtR with osteoporosis.

WHtR	Crude model		Adjusted model^+^	
	OR (95%CI) ^a^	*p*-value	OR (95%CI) ^a^	*p*-value
<0.57	0.41 (0.26,0.65)	<0.001	0.50 (0.31,0.82)	0.007
≥0.57	0.83 (0.63,1.10)	0.199	0.86 (0.63,1.16)	0.316

WHtR, waist to height ratio.

a WHtR was entered as a continuous variable per increase 0.1.

OR, odds ratio; CI, confidence interval.

+Adjusted for age, gender, race, poverty income ratio (PIR), educational level, smoking status, drinking status, cardiovascular disease (CVD), hypertension, diabetes mellitus (DM), hemoglobin A1c (HbA1c), alanine transaminase (ALT), aspartate transaminase (AST), serum total bilirubin, serum creatinine, uric acid, blood urea nitrogen (BUN), serum sodium, serum phosphorus, total calcium, serum potassium, serum iron.

### 3.4 Subgroup analyses

**Figure 3:**
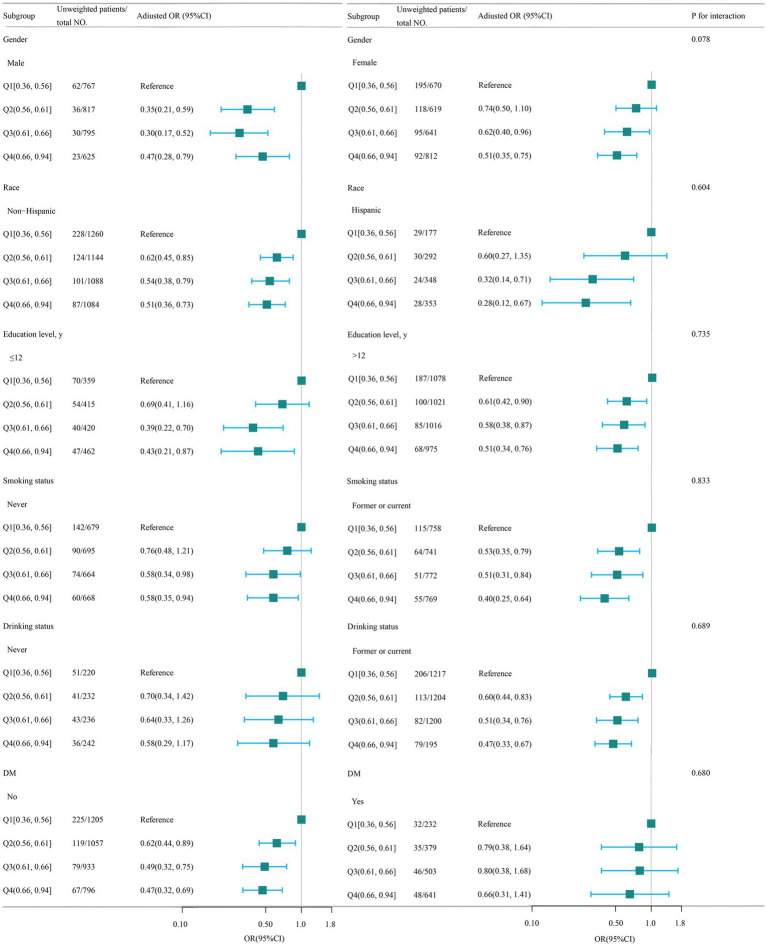
Association between WHtR and OP according to the general characteristics. WHtR, waist to height ratio; OP, osteoporosis. Except for the stratification factor itself, the stratifications were adjusted for all variables (age, gender, race, poverty income ratio, educational level, smoking status, drinking status, cardiovascular disease (CVD), hypertension, diabetes mellitus, hemoglobin A1c, alanine transaminase (ALT), aspartate transaminase (AST), serum total bilirubin, serum creatinine, uric acid, blood urea nitrogen (BUN), serum sodium, serum phosphorus, total calcium, serum potassium, serum iron). Squares indicate odds ratios (ORs), with horizontal lines indicating 95% CIs.

A stratified analysis was conducted in many subgroups to evaluate possible impact alterations on the association between WHtR and OP. There were no notable interactions seen in any subgroups when analyzing the data based on gender, race, education level, smoking status, drinking status, and DM (Figure 3).

### 3.5 Sensitivity analysis

The link between WHtR and OP remained consistent after adjusting for the covariate of vitamin D. A lower WHtR in Q1 (≤0.36 to ≤0.56) was linked to a higher risk of OP. The adjusted odds ratio (OR) for WHtR and OP was 0.47 (95% CI: 0.32–0.70, *p* < 0.001) in Q3 (<0.61 to ≤0.66) and 0.52 (95% CI: 0.36–0.76, *p* = 0.001) in Q4 (<0.66 to ≤0.94) (Table 5).

**Table 5:** Association between WHtR and osteoporosis after adjusting for the covariate of vitamin D.

WHtR	No.	Crude OR (95%CI)	*p*-value	Model 1 OR (95% CI)	*p*-value	Model 2 OR (95% CI)	*p*-value	Model 3 OR (95% CI)	*p*-value
Q1 [0.36, 0.56]	1,152	1 (Ref)		1 (Ref)		1 (Ref)		1 (Ref)	
Q2 [0.56, 0.61]	1,152	0.68 (0.49, 0.94)	0.019	0.78 (0.56, 1.09)	0.141	0.76 (0.53, 1.09)	0.133	0.78 (0.54, 1.13)	0.184
Q3 [0.61, 0.66]	1,152	0.44 (0.31, 0.63)	<0.001	0.51 (0.35, 0.74)	<0.001	0.48 (0.33, 0.70)	<0.001	0.47 (0.32, 0.70)	<0.001
Q4 [0.66,0.94]	1,153	0.46 (0.33, 0.63)	<0.001	0.47 (0.34, 0.66)	<0.001	0.52 (0.37, 0.74)	<0.001	0.52 (0.36, 0.76)	0.001
Trend test			<0.001		<0.001		<0.001		<0.001

WHtR, waist to height ratio; Q, quartiles; OR, odds ratio; CI, confidence interval; Ref: reference.

Crude: no other covariates were adjusted.

Model 1 was adjusted for gender, uric acid.

Model 2 was adjusted for model1 + age, race, poverty income ratio (PIR), educational level, smoking status, drinking status, diabetes mellitus (DM), hemoglobin A1c (HbA1c), alanine transaminase (ALT), serum total bilirubin, serum phosphorus.

Model 3 was adjusted for model2 + cardiovascular disease (CVD), hypertension, aspartate transaminase (AST), serum creatinine, blood urea nitrogen (BUN), serum sodium, total calcium, serum potassim, serum iron, VitD.

## 4 Discussion

This cross-sectional study employed data from 5 cycles of NHANES to analyze the relationship between OP and WHtR in the U.S. elderly population. The findings, after controlling for potential confounding variables, established a negative correlation between WHtR and the occurrence of OP. Notably, restricted cubic spline (RCS) analysis showed that WHtR and OP prevalence had a nonlinear inverse relationship. A threshold was found at WHtR = 0.57 using threshold effect analysis. An increase in WHtR within a certain range was associated with a reduced risk of OP. Subgroup analyses further confirmed the stability of this inverse correlation across different populations. Ultimately, sensitivity analysis reinforced the robustness and consistency of the research outcomes.

Prior research has assessed the correlation between obesity and OP, or bone density, using several anthropometric measures. The BMI is a commonly used metric to assess obesity. However, as research has progressed, the obesity paradox has emerged, raising questions about the accuracy of BMI as a measure of obesity among researchers (48, 49). The reason is likely that BMI only assesses general obesity and disregards body fat distribution (50). Nevertheless, several investigations have shown a strong correlation between the allocation of fat and bone metabolism, particularly in the case of abdominal obesity (51, 52). WC, WHR, and WHtR are measurements used to evaluate the distribution of abdominal fat. These measurements have been used in several studies to examine the relationship between central obesity and bone health (4, 5). Prior research has examined the connections between WC or the WHR and BMD or OP. However, the results have been inconclusive, mostly due to the significant effects of gender, age, and race (8). The WHtR has some benefits when compared to other measures of central obesity, such as WC or WHR. WHtR is less impacted by factors such as sex or ethnicity (7). Moreover, our investigation reveals substantial variations in biochemical markers across different WHtR groups, indicating the effectiveness of WHtR as an indicator of central obesity and its significance for hepatic and renal metabolic profiles. This aligns with the findings of Huang et al., who found that WHtR serves as an independent and superior predictor of hyperuricemia (53). Chen et al.’s study also supports our findings, identifying WHtR as the most effective anthropometric measure for assessing adult ALT levels (54). These converging findings highlight the significance of WHtR in assessing the risk of obesity-related metabolic diseases. In addition, the most current United Kingdom obesity recommendations (55) have advised using WHtR as a marker of obesity. Therefore, the WHtR is a reliable metric used to evaluate the correlation between obesity and OP or BMD.

Tian et al. conducted a study in 8457 people using quantitative heel ultrasonography (QUS) to quantify BMD. They then examined the relationship between WHtR, BMD, and OP. The researchers determined that an increase in WHtR was strongly linked to greater estimated bone mineral density BMD and a reduced risk of OP. Furthermore, they noted that the older participants appeared to be more inclined to experience the fat-protect-bone effects (4). Nevertheless, several studies have shown a detrimental correlation between WHtR and BMD (5, 56). Jongseok Lee et al. examined the relationship between WHtR and BMD using data from 2060 Korean adolescents aged 10–19 years. The data was obtained from the Korea National Health and Nutrition Examination Survey (KNHANES), performed between 2008 and 2011. Their findings indicate a nonlinear “inverted *U*-type” correlation between WHtR and BMD (57). In addition, the existing research indicates that a greater WHtR is linked to a higher occurrence of hip fracture and spine fracture (58, 59). The current research used multivariable regression analysis to account for any confounding factors and ensure the generalizability of the findings to the elderly population in the United States. This approach differs from earlier studies. Our dose–response study revealed that there is a non-linear connection between WHtR and OP, specifically an *L*-type relationship with an inflection point value of 0.57. Within the specified range, the WHtR has a negative correlation with OP. However, once the ratio surpasses the specified threshold, this correlation ceases to exist. This outcome serves as a helpful addition to the existing body of research. Hence, we hypothesize that when the WHtR is less than 0.57, changes in variables such as mechanical stress and hormone may have a good influence on bone health. However, when the WHtR is equal to or greater than 0.57, the bad effects of belly fat buildup, changes in body composition, inflammation, insulin resistance, and other things may cancel out the good effects on bones that have already been shown. Furthermore, the connection remained strong and consistent in sensitivity and subgroup analysis.

The global prevalence of obesity has increased significantly and rapidly in recent decades, reaching epidemic levels (60). Defined as a medical condition characterized by excessive fat accumulation, obesity reduces life expectancy and is associated with various illnesses such as type 2 diabetes mellitus, hypertension, cardiovascular disease, depression, cancer, and OP (61). While several recent studies have explored the link between obesity and OP, the relationship between obesity and bone metabolism remains complex and somewhat unclear. Here are some potential mechanisms to consider: Obesity may impact bone metabolism through various factors, including mechanical stress, hormones, adipokines, cytokines, and other elements. This influence can be broadly categorized into positive and negative effects. On the positive side, obesity can increase bone density to cope with heightened mechanical stress (62). Elevated fat content is associated with increased estrogen production, which promotes the growth of osteoblasts and inhibits bone breakdown (63, 64). On the other hand, obesity increases the risk of insulin resistance, which may lead to a reduction in the differentiation and multiplication of osteoblasts and an increase in the production of osteoclasts (65–69). Additionally, obesity may lead to systemic low-level inflammation, resulting in increased osteoclast activity and bone breakdown (61, 70). Besides, leptin and adiponectin are the main adipokines secreted by adipose tissue. Obesity may elevate leptin levels and reduce adiponectin levels, which has been demonstrated to enhance osteoclast activity and result in bone loss (71). Furthermore, obesity has been shown to stimulate the transformation of bone marrow stem cells into fat cells, leading to a decrease in bone-forming cells in the bone marrow (70, 72, 73). Additionally, studies have found that obese individuals have lower levels of vitamin D, a crucial osteotropic factor, potentially due to factors such as dilution in body fluids, storage in fatty tissue, reduced sunlight exposure, and decreased production in the body (74).

This research has several constraints. Primarily undertaken among an elderly American demographic, more investigation is required to ascertain the applicability of our results to other groups. Also, even when regression models, stratified analysis, and sensitivity analysis are used, it is not possible to completely rule out the effect of unknown or unmeasured variables, which may still cause residual confounding effects. Furthermore, we did not account for BMI as a covariate since there was a significant collinearity between WHtR and BMI. Additionally, due to limitations in sample selection, we were unable to directly compare the associations of BMI, WC, and WHR with osteoporosis within the same cohort. This restriction may have affected the generalizability and comparability of our findings. Consequently, our results may not fully represent the true relationships between these indices and osteoporosis. This limitation could have impacted our comprehensive assessment of osteoporosis risk factors. Given these constraints, future studies should consider conducting more in-depth analyses of the relationships between these obesity indices and osteoporosis within a single sample to provide a more comprehensive understanding. Ultimately, because of the inherent constraints of cross-sectional research, it is not possible to establish a definitive cause-and-effect connection between WHtR and OP. Therefore, it is necessary to conduct further longitudinal studies to validate this link.

## 5 Conclusion

In conclusion, this study indicates that the association between WHtR and OP was not linear, exhibiting a point of inflection at around 0.57. Further prospective studies are required to investigate the correlation between WHtR and OP in the future. Additional research, particularly long-term studies, would be essential to confirm these results and investigate their mechanism of action.

## Data Availability

Publicly available datasets were analyzed in this study. This data can be found at: http://www.cdc.gov/nchs/nhanes.htm.

## Footnotes

^1^http://www.cdc.gov/nchs/nhanes.htm, last accessed on May 1, 2024.

^2^ http://www.R-project.org

^3^ http://www.clinicalscientists.cn/freestatistics
